# Qualitative analysis of the factors associated with whistleblowing intentions among athletes from six European countries

**DOI:** 10.3389/fspor.2024.1335258

**Published:** 2024-05-07

**Authors:** John Toner, Luke Jones, Lucas Fairs, Constantine Mantis, Vassilis Barkoukis, Garyfallia Daroglou, John L. Perry, Andrei V. Micle, Nikolaos C. Theodorou, Sabina Shakhverdieva, Marius Stoicescu, Constantin Pompiliu-Nicolae, Milica V. Vesic, Nenad Dikic, Marija Andjelkovic, Jesús Muñoz-Guerra Revilla, Elena García-Grimau, Miguel A. E. Martínez, Javier A. Amigo, Anne Schomöller, Adam Robert Nicholls

**Affiliations:** ^1^ School of Sport, Exercise and Rehabilitation Sciences, University of Hull, Hull, United Kingdom; ^2^ Department for Health, University of Bath, Bath, United Kingdom; ^3^ School of Social Sciences, Humanities & Law, Teesside University, Middlesbrough, United Kingdom; ^4^ Department of Physical Education and Sport Sciences, Aristotle University of Thessaloniki, Thessaloniki, Greece; ^5^ Department of Physical Education and Sport Sciences, University of Limerick, Limerick, Ireland; ^6^ Romanian National Anti-Doping Agency, Bucharest, Romania; ^7^ Independent Researcher, Athens, Greece; ^8^ Faculty of Physical Education and Sport, National University of Physical Education and Sport, Bucharest, Romania; ^9^ Anti-doping Agency of Serbia, Belgrade, Serbia; ^10^ Sports Medicine Associations of Serbia, Belgrade, Serbia; ^11^ Comisión Española para la Lucha Antidopaje en el Deporte (CELAD), Spain; ^12^ International Council of Sport Science and Physical Education, Berlin, Germany

**Keywords:** doping, intervening events, fairness norms, loyalty norms, whistleblower

## Abstract

Although whistleblowing is thought to represent an effective mechanism for detecting and uncovering doping in sport, it has yet to become a widely adopted practice. Understanding the factors that encourage or discourage whistleblowing is of vital importance for the promotion of this practice and the development of pedagogical material to enhance the likelihood of whistleblowing. The current study employed a qualitative methodology to explore the personal and organisational factors that underpin intentions to blow the whistle or that may lead to engagement in whistleblowing behaviours in sport. Thirty-three competitive athletes across a range of sports took part in a semi-structured interview which sought to explore what they *would do* should they encounter a doping scenario. Content analysis revealed that whistleblowing is a dynamic process characterised by the interaction of a range of personal and organisational factors in determining the intention to report PED use. These factors included moral reasoning, a desire to keep the matter “in-house”, perceived personal costs, institutional attitudes to doping, and social support. Analysis revealed a number of “intervening events”, including a perceived lack of organisational protection (e.g., ethical leadership) within some sporting sub-cultures, which present an important obstacle to whistleblowing. The intention to report doping was underpinned by a “fairness-loyalty trade-off” which involved athletes choosing to adhere to either *fairness* norms (which relate to a sense that all people and groups are treated equally) or *loyalty* norms (which reflect preferential treatment towards an in-group) when deciding whether they would blow the whistle. The promotion of fairness norms that emphasise a group's collective interests might encourage athletes to view whistleblowing as a means of increasing group cohesiveness and effectiveness and thereby increase the likelihood of this practice.

## Introduction

In December 2014, Yuliya and Vitaliy Stepanov appeared in a documentary on a German TV network and blew the whistle on systemic doping within the Russian sports system. Fearing reprisal from the Russian state, the couple fled their homeland and sought sanctuary in Germany where they detailed their doping accusations (2). Any concerns they may have had over their safety were confirmed in 2016 when the World Anti-Doping Agency (WADA) revealed that Yuliya's athlete account (which contained information of her whereabouts) had been hacked. In a subsequent investigation, WADA determined that Yuliya was the only athlete to have had her details compromised in this manner. Whistleblowing is touted as an important means of tackling corruption and wrongdoing in sport but whistleblowers across a range of domains have been found to face recrimination after speaking up (3–5). As the Stepanovs' experience illustrates, athletes may be fearful of retaliation should they blow the whistle whilst others might be reluctant to contravene the “code of silence” that governs practice in this field (6, 7). There is a growing recognition by WADA and national doping agencies (NADO's), however, that more needs to be done to provide platforms that allow athletes to report doping-related irregularities in a manner that protects them against potential recrimination (8).

The use of performance enhancing drugs (PEDs) and other illicit substances is a major problem in contemporary sports culture. Indeed, doping is seen to represent a threat to the integrity of sport by undermining the key principles of open and fair competition (9). The prevalence of PEDs is also cause for concern given the deleterious impact they are known to have on users' physical and mental health (10). Anabolic Androgenic Steroids, for example, have been found to account for 43% of doping offences in grassroots sport, and are associated with serious ill effects on the liver, heart, kidneys, and reproductive systems (11, 12). Unfortunately, little is known about the true prevalence of doping in sport (13). Although WADA (14) typically report that 1%–2% of athletes commit doping offences on a yearly basis, recent research has shown that up to 57.1% of elite athletes may have been involved in a doping offence in the past (see (15) and (16). The latter evidence calls into question the efficacy of current drug testing regimes and suggests that current efforts to deter and/or identify doping behaviour are proving ineffective.

An alternative approach to identifying doping behaviour is to encourage athletes, coaches, or sporting personnel to blow the whistle on doping offences (9). According to WADA, whistleblowing against doping is defined as the disclosure of sensitive information about athletes and their entourage with respect to anti-doping rule violations (ADRVs); World Anti-Doping Code non-compliance violations; and acts or omissions that could undermine anti-doping efforts (17). Whistleblowing has the potential to result in the investigation, detection, prosecution of anti-doping rule violations, which may have otherwise gone undetected (17). As such, whistleblowing may represent an effective way of deterring doping in sport. In seeking to promote whistleblowing, WADA's revised code [Article 10.6.1 (18);] offers reduced sanctions to those athletes who provide information that might lead to an ADRV. As already discussed, however, the decision to blow the whistle is a complex one that is influenced by factors such as whether one considers it one's responsibility (19), the nature of the wrongdoing (20), and the consequences one might face from lifting the lid on corruption (21).

## Theoretical framework

Unfortunately, very little is known about the factors that predict whether athletes, coaches, and other sporting personnel (e.g., doctors, physiotherapists, nutritionists) will blow the whistle on a doping offence or remain silent when they witness or become aware of doping violations. By contrast, in the wide ranging and extensive literature on doping in sport, the Theory of Planned Behaviour [TPB (1)] is commonly drawn upon to predict athletes' intention to dope [e.g. (22)]. According to this theory, behavioural intentions are influenced by a combination of *attitudes* (i.e., positive or negative evaluations of performing a specific behaviour), *perceived behavioural control* (i.e., one's evaluation about their capabilities to perform a behaviour), and *subjective norms* (i.e., the pressure individuals perceive from others to engage in behaviour). The TPB has recently been extended to investigate the relationship between morality and doping, given that doping is considered an immoral behaviour. Following others, the current study draws upon the TPB framework to identify the factors that influence the intention to whistleblow in sport.

We should note, however, that TPB is a rationalistic model that may not fully account for the cognitive, affective, or situational factors that influence behaviour. Indeed, evidence on whistleblowing within government agencies reveal that a complex interaction of perceived personal costs and organisational factors contributes to whether people will report corruption or turn a blind eye (23). In Whitaker et al.'s (24) study, rugby league players admitted that the code of silence that permeates their sub-culture would prevent them from reporting doping behaviour. Erickson et al. (6) found that student-athletes were prepared to confront PED users but reluctant to blow the whistle about this practice. Further research from Erickson et al. (25) showed that student-athletes would be most likely to address PED use via confrontation. This evidence suggests that some athletes might consider the betrayal of a fellow athlete to be a more egregious act than a failure to disclose their knowledge of doping behaviour to the authorities. These findings point to the complex nature of the whistleblowing process and the moral dilemma faced by athletes who may wish to remain loyal to their teammates on the one hand but adhere to the sporting principle of fair play on the other (27).

In the organisational literature, the preceding moral dilemma has been described as involving a “fairness-loyalty trade-off” (18) whereby individuals choose to adhere to either fairness norms (which relate to a sense that all people and groups are treated equally) or loyalty norms (which reflect preferential treatment towards an in-group) when making decisions about their behaviour. Erickson et al.'s (26) interviews with three athletes who had each blown the whistle on doping in a sporting context corroborate this moral dilemma. The athletes in this study did not immediately report their knowledge of doping owing to a sense of guilt in betraying teammates and a concern in jeopardising loyalty (i.e., by adhering to fairness norms). Erickson et al.'s (26) findings represent the first within the whistleblowing in sport literature to evidence the fairness-loyalty trade-off and, importantly, the emotional distress faced by those who choose to report doping. The fairness-loyalty trade-off might also be complicated by organisational perceptions of unethical behaviour (e.g., the extent to which the sporting sub-culture is opposed to doping) and the level of protection afforded to whistleblowers (17). In Latan's et al.'s (27) study, for example, predicted organisational protection had a significant positive effect on whistleblowing intention amongst employees working for a government agency in the United States but not amongst employees in the emerging economy of Indonesia (due to a weakness of WPA). Organisations that provide employees with high levels of protection may increase the intention to engage in whistleblowing. The intention to whistleblow is also shaped by contextual factors related to one's organisation (e.g., whether an organisation educates its employees about channels for reporting unethical behaviours) and situation (e.g., fear of retaliation). However, research has yet to explore how organisational and personal factors might interact to prevent or encourage whistleblowing in sporting contexts. The current study will draw on Waytz et al.'s (18) notion of a fairness-loyalty trade-off to explore how organisational endorsement of fairness over loyalty, or vice-versa, might influence an athlete's intention to report wrongdoing. Understanding how these factors might encourage or discourage whistleblowing is vital for the promotion of this practice and the development of pedagogical material to enhance the likelihood of whistleblowing. Thus, this study sought to extend current understanding by conducting interviews with athletes from six European countries to explore how organisational and personal factors interact to promote or deter whistleblowing in sport.

## Methodology

### Philosophical orientation

The study was grounded in a post-positivist paradigm (28) which has implications for both our ontological (i.e., critical realism) and epistemological stance (i.e., modified dualist/objectivist). Adopting this ontological position means that we consider there to be an objective reality within the social world, whilst acknowledging that any examination of this world is inevitably value laden and shaped by context. Nevertheless, the application of methodological rigor and analysis might allow us to reach an approximation of the truth. Our paradigmatic stance has implications for the study including our choice of method (i.e., interviews that were informed by existing literature and standardised across participants), data collection (i.e., single interviews), data analysis (e.g., a deductive approach using categories derived from theory), trustworthiness techniques (e.g., peer debriefing) and how we choose to represent the findings (i.e., realist form characterized by experiential authority, the participant's point of view and conveying interpretive omnipotence).

### Participants

Given the difficulty in recruiting athletes who have engaged in whistleblowing behaviour, purposive sampling was used to identify and select individuals from a range of countries and sports who were familiar with the whistleblowing process. None of the recruited participants had previously engaged in whistleblowing. Some project members drew upon their federation's anti-doping database to send email or Whatsapp invitations to participants. Other participants were recruited via an email invitation disseminated to local sports clubs and University students and some participants were recruited through our personal and professional networks. Each participant who met the inclusion criteria (i.e., a competitive athlete over 18 years of age) and expressed an interest in participation was informed of the study's purpose and what it would entail. The sample consisted of 19 males and 14 females aged between 18 and 40 years (M_age_ = 24.78, SD = 4.75). All participants were competitive athletes from a diverse range of sports including basketball, football, and triathlon (see Table 1 for a more detailed overview of participant demographics). Nine participants were recruited from Greece, six from Spain, three from Romania and five participants each were recruited from the United Kingdom, Germany, and Serbia. Ethical approval was granted by the University ethics committee and all participants provided informed consent prior to data collection.

**Table 1:** Participant demographics.

Name (Pseudonym)	Gender	Sport	Level
Scott	M	Powerlifting	International
Marcus	M	Basketball	National
Mike	M	Canoe Polo	International
Paul	M	Duathlon	International
Amelia	F	Field Hockey	National
Luke	M	Triathlon	International
Lee	M	Modern Pentathlon	International
Andrea	F	Long Distance Running	International
Katrina	F	5,000 m Runner	International
Oscar	F	Pole Vault	International
Agnes	F	Artistic Gymnastics	International
Nicholas	M	Pole Vault	International
Adrian	M	Soccer	National
Henry	M	Long Jump	National
Maisie	F	Volleyball	National
Agatha	F	Swimming	National
Angelica	F	Handball	National/Regional
Rory	M	Soccer	B Amateur
Ronnie	M	Long Jump	National
David	M	Athletics	International
Ava	F	Shooting	International
Sophia	F	Kayak Canoe	International
Charlotte	F	Kayak Canoe	International
Eliza	F	400 m	National
Noah	M	Rugby	International
Samuel	M	Weightlifting	International
Mia	F	3,000 m steps	International
William	M	20 km race walking	International
Alan	M	Triathlon	National
Emma	F	Para-Cycling	International
Ryan	M	Football	National
Dylan	M	Football	National
Ollie	M	Tennis	International

### Data collection

This study was part of a larger cross-cultural project seeking to understand and promote whistleblowing on doping irregularities in the European Union. Participants took part in a semi-structured interview held via one of the following online platforms: Zoom, Skype, Microsoft Teams, and BlueJeans. The interview schedule was informed by existing literature on whistleblowing [e.g., (9, 18, 26)] and the Theory of Planned Behaviour. The interview began by providing participants with a definition of both doping and whistleblowing. Interviewees sought to develop rapport with participants by assuring them that responses would remain anonymous and briefly discussing their sporting background. Next, participants were provided with a description of the following scenario: “One day, while sitting in your club's changing room, you overhear a teammate admitting that he or she has used steroids and other performance enhancing drugs for the last three months to two other teammates. You then see him/her showing these teammates the drugs in different containers. During this chat, the athlete tells the two teammates that he/she is able to lift heavier weights, feels more powerful in competition, and recovers faster while using these substances. After talking about the benefits, the teammate tells the other athletes where they can get the steroids from and that it will help their performance too”. A scenario was presented in this manner to contextualise whistleblowing and to provide participants with a concrete example that would aid their reflections and stimulate discussion over the course of the interview. The remainder of the interview sought to explore how a variety of psychological, personal, and organisational factors would influence the participants’ intention to whistleblow. Interviews were semi-structured in nature which meant that while participants were asked the same questions in the same way (e.g., How do you feel your personal relationship with the teammates in the group might influence the intentions to report the incident? How do you feel the club/organisation's culture might affect your intentions to report the incident?), the sequence of questions varied according to the flow of the conversation and follow-up probes (e.g., “Can you explain in more detail how this factor would influence your intention to report a doping offence?”) were used to encourage more elaborate and in-depth responses. The interviews closed by providing interviewees with an opportunity to discuss any topics they deemed relevant to whistleblowing. Interviews lasted an average of 35 min (range between 25 and 50 min) and were transcribed verbatim. Interviews were either conducted in English or in the participants' native language and translated by the interviewer into English prior to analysis.

### Data analysis

Following transcription, data were analysed using a directed approach to content analysis (29). First, transcribed interviews were read several times to gain a clear comprehension of the participants' responses and then subjected to line-by-line analysis. This process involved segmenting sentences from the interview transcripts into analytical memos that encompassed the participants' opinions regarding factors that would influence their intention to whistleblow. Initially, this was a deductive approach using pre-identified theory (e.g., TPB) to assign codes, and their operational definitions, to each chunk of text identified as being relevant to the study. This was followed by an inductive approach that involved assigning a new code to any text that could not be categorised using the pre-determined theoretical frameworks. Codes were subsequently grouped together to form emergent categories (lower-order themes) based on their similarity to each other and distinction from other categories (30). This process was then repeated to generate higher-order themes. The construction of themes involved a manifest analysis (i.e., a deductive or top-down approach whereby understanding is derived from a literal and theory-informed reading of words) followed by latent analysis (i.e., an inductive or bottom-up analysis that seeks to uncover implicit meaning).

### Trustworthiness

Trustworthiness refers to the level of trust regarding the procedures that ensure that data is appropriately and ethically collected, analysed, and reported (31). In line with our epistemological stance, we employed two techniques to maximise the trustworthiness of our data. First, peer-debriefing was conducted throughout the data analysis procedure by the research team, who provided guidance, critical evaluation, and sought to challenge the primary researcher's interpretations (32). Peer-debriefing occurred via telecommunications, e-mails, and on-line meetings between the primary researchers and other researchers. The aim of this process was to establish a general agreement between the researchers in terms of how the data was being coded and not for the purposes of establishing inter-rater reliability (33). Two of the researchers identified themes independently before acting as critical friends and questioning each other's interpretations (33). The second technique to enhance trustworthiness involved asking a third researcher to cast a critical eye over the results, after the analysis had been completed (34). The researcher encouraged the research team to reflect upon and explore alternative interpretations of the data.

## Results

It is important to note that findings illustrate what participants *would do* should they encounter a doping scenario and are not necessarily illustrative of actual behaviours or experiences. The themes are presented in a chronological order which represents the most common sequence of steps taken by participants when deciding whether to whistleblow or not. Pseudonyms have been used to protect the participants' anonymity. The themes include moral reasoning, keeping the matter “in-house”, perceived personal costs, team climate, social support, and facilitating factors which included assurances and anonymity (see Table 2). We start by considering how moral reasoning influences the intention to whistleblow.

**Table 2:** Higher and lower-order theme*s.*

High-order themes	Lower order-themes	Frequency
Moral reasoning	Unethical practice	30
	Spirit of sport	27
	Reputation of sport	15
Keeping matters in-house	Confrontation	20
	Code of silence	12
Perceived personal costs	Fear of retaliation	11
	Damage relationships	10
Team climate	Institutional attitudes	20
	Social support	18
Facilitating factors	Assurances	12
	Anonymity	11

### Moral reasoning

The general consensus amongst participants was that doping is unethical and that it contravenes the spirit of competitive sport. Many of the participants are deeply opposed to the practice and felt duty bound to blow the whistle on perceived doping offences. Illustrating this, Scott stated:

*From my own personal view, it would be that I couldn't live with myself knowing that I'm lying or helping because I would then put myself at risk of breaching. Or if I didn't say something I would be perjuring myself in a legal sense with an ADO. So ethically I would have to say something regarding it and morally I would be obliged because it's not part … It's not how I was raised and I feel I'd be letting not only myself down but my family and friends as well*.

Consistent with findings from previous whistleblowing studies [e.g., (6),], it was clear that participants felt that certain standards should guide human cooperation within sport. In particular, many athletes had an unfavourable attitude and subjective norm (1) with respect to doping. David considered doping “bad for fair play” whilst Luke noted that his values “are deeply rooted. I say it's wrong and has nothing to do with fair sport. That's why I take actions against it” whilst Andrea stated that her opposition to doping was all “about respect towards the reason why we are all doing sports. I am against it and this is why it is important to me to uncover such injustice”. For these athletes, their decision to report wrongdoing was underpinned by an adherence to *fairness* norms or a sense that all people and groups are treated equally (18). It also seems that these athletes are highly motivated to address wrongdoing because of the strength with which they identify with the offending ingroup, and their belief that doping represents a violation of the group's own values (35).

Those morally opposed to doping were also concerned that their sport's image might be tarnished should several athletes be subject to ADRVs. These athletes felt an obligation to uphold principles of fairness. Oscar noted that he has a “responsibility for my discipline, my sport, and also for the sports club and on the other side for this person … that a healthy life is secured” whilst Lee indicated that he “would prefer that one hears pentathlon and thinks of medals, instead of a doping scandal”. These athletes wished to preserve the integrity of their sport—something they may see as important given the reputational damage that sport has suffered as a result of ongoing doping controversies. All participants were fundamentally opposed to the use of PEDs and considered this behaviour to be hugely problematic and to violate the spirit of sport. Thus, one might assume that they would consider blowing the whistle to be an essential process in the preservation of clean sport. However, our findings paint a more complex picture and suggest that the decision to blow the whistle either inside or outside their organisation is influenced by the interaction of a number of personal and organisational factors. Athletes might have a favourable attitude towards whistleblowing but the decision to engage in this practice is influenced by a range of “intervening events” (1) that produce changes in their intentions or sense of perceived behavioural control.

### Keeping the matter “in-house”

Many of the participants admitted that their first course of action upon learning of doping would be to confront the suspected user directly. Some participants acknowledged their reluctance to go straight to the authorities with a complaint and would much prefer to engage the athlete in question. As Marcus noted:

*So I would never try to be a person that snitches, especially on a teammate because it is some sort of a family, a brotherhood that you create with people that you don't perhaps know even outside the court and yet you have to trust with your life when you're on the court … I'll probably would never even start whistle blowing to the coach or whomever in the sports club administration before I would approach a player or the group of players that I’m concerned about*.

Instead of going to the authorities, many participants remain loyal to their teammates by keeping the matter “in-house”. Echoing findings from Erickson et al.'s (6) study, the athletes would confront their teammates in the hope that they might change their behaviour and re-consider their decision to take PEDs. Marcus further revealed that he “would never report on the person as long as I would do everything in my power to talk him through it to make him stop and to help him or her deal with those kind of issues”. Several participants were perfectly aware, however, that confronting a teammate about suspected PED use is unlikely to bring about a swift resolution to the matter. Take, for example, Katrina's acknowledgement that the way the athlete responds to a doping confrontation would have a significant bearing on the whistleblowing process: “if they were good friends and they would say “nonsense, it does not matter” I would have to think about it again”. Scott expressed a similar sentiment:

*I'd probably speak to the guy who's got the drugs on his own and the other two, either as a group or two of them on their own and say “have you thought about what would happen to the rest of the team, if you were to get caught?*

Scott admits, however, that he would “have to actually see how other players and the team react”. Here we see evidence that intentions will only find expression in whistleblowing behaviour if athletes perceive the behaviour to be under volitional control (1). Many athletes in this study have a sense that a successful outcome to whistleblowing (i.e., termination of doping) is beyond their control. As such, if a suspected doper reacted adversely to a confrontation then some athletes would be unlikely to pursue the issue much further. A negative reaction would act to remind the whistleblower of the subjective norm or expectation that they remain silent about their discovery.

Participants noted how the culture of silence that exists within their sport would compel them to turn a blind eye to doping. In this respect, Angelica explained how she “would not do it [report a doping incident] because there is an informal rule that says that whatever we say in the locker room stays in the locker room and does not come out”. Whistleblowing may not be the subjective norm within certain sporting sub-cultures and these athletes may feel a perceived social pressure not to perform the behaviour (1). Instead, there was a clear preference amongst these participants for keeping such matters “in-house” in the hope that it could be dealt with without the need to involve the authorities. This is particularly the case where there is high level of cohesion within the team or if the athlete happened to have a close relationship with the suspected doper. Indeed, Marcus conceded that “if I have a strong cohesion with teammates, I’ll be less likely to report it externally … the more support I have for resolving the matter internally, the less probable I am to report the matter externally”. When expressing her concern about how doping revelations might disrupt the social bonds within her own team, Katrina noted that “I think I would first talk to others about it and then decide “let us do something against it” … I would also check if I destroy my environment/climate”.

Although the athletes expressed a preference for confronting their teammate(s), they were clearly uneasy about this process. Paul suggested that “if it was your best friend, it would be difficult and it would probably be something for you to discuss and try to discourage before they went down the route of doping”. This finding mirrors the “internal struggle” faced by whistleblowers in Erickson et al.'s (26) study who were forced to wrestle with the thought of whether reporting doping was worth it or not. Paul admitted, however, that he would not proceed to blow the whistle if the initial confrontation failed to put an end to doping behaviour. Similarly, Scott argued that if the suspected doper was a:

*captain or well-known figure or legend within this team or club, I think you'd be very reluctant for that information to come out … If you are like best mates, teammates, sharing rooms, training partners…it's gonna make it really hard [to blow the whistle]*.

Athletes who were fearful about potential retribution would seek more information about the suspected doping case before confronting the athlete as noted by Andrea:

*I think one should ask that person what is going on. I think I would be a bit afraid … I mean depending on the result … how far will this go, you know* … *if I knew the outcome for that person … that's an important question … I would not immediately do something. I would first investigate … I would clarify the situation*.

The finding that some athletes are reluctant to blow the whistle externally on their teammates is not entirely surprising [see (18) and (6) for similar results]. The decision to keep matters “in-house” and not report doping behaviour externally was driven by the athlete's sense of loyalty to their teammates or training partners. Waytz et al. (18) argue that the “fairness-loyalty” quandary is likely to be heightened if the whistleblower and the PED user have a close relationship and this is evident in our findings. Many of our athletes were concerned that whistleblowing might result in them being ostracized from their respective in-groups—a process which is associated with a significant decline in an individual's fundamental needs, mood, status, and competence (36). Sports teams are often characterised by close social bonds and athletes may be reluctant to do anything that threatens group cohesion or undermines a friendship forged over many years. It would appear that whilst the athletes are prepared to directly confront the PED user, many would be hesitant to report doping behaviour given their concerns over how this might influence team dynamics or their own standing within the setting. The likelihood that they go beyond this initial confrontation with the suspected doper by reporting their concerns to the authorities depends on a number of personal and organisational factors. The next theme addresses the perceived personal costs faced by those who may choose to blow the whistle.

### Perceived personal costs

The reluctance to report a doping offence was influenced by a number of perceived costs including the fear the athlete might face retribution or retaliation. Many were morally opposed to doping but expressed concern about what they might lose should their identity as a whistleblower be uncovered. The fear of reprisal was expressed by many participants who were aware of the perceived expectations or subjective norms of others (1) within their sporting sub-culture. For instance, Luke indicated that within his club there are:

*hierarchies and there is the danger that you harm yourself/shoot yourself in the foot … you could be in trouble because the athlete is maybe the best in the club and holds a good position within the club … and his position is so strong … for a club commission … this could have disadvantages for oneself or that the information will not be forwarded*.

Other participants were afraid of how whistleblowing might affect their standing within the team. Paul also admitted that:

*You don't want to cause rifts in the team, which could result in you being sort of excluded from the team or sort of made to feel not valued I guess. You don't want to be ostracised from all of your peers that you've spent months or years training with*.

The fear that reporting wrongdoing will result in “ostracism” mirrors the findings on whistleblowing in professional football [see (37)]. Mike expressed such concerns when he revealed that:

*if I lose that social relationship with the person who has been caught do I then lose the trust of my teammates? And I know again it's not right. That's not the way it should be but that's just the way society works and that's the morals and values of some people are very different to mine obviously*.

### Team climate

The higher order theme “team climate” included the lower order themes “institutional attitudes to doping” and the “social support” athletes received.

### Institutional attitudes to doping

The athletes' intention to whistleblow externally was heavily influenced by the extent to which their organisation values and supports whistleblowing as a process. Additionally, if a club/organisation actively promote anti-doping values then athletes appear more likely to blow the whistle. In this respect, Paul noted that:

*If there was a strong anti-doping policy and they were quite vocal about it and quite supportive of raising those issues, then I would definitely be more inclined then if I was aware that they had no intention to raise an issue … if raising doping allegations wasn’t valued then you'd say well what's the point because it will fall on deaf ears*.

A number of athletes had less fear of personal costs perhaps owing to a sense that they have the opportunities (i.e., reporting channels) and resources (e.g., cooperation of senior stakeholders) to ensure a successful outcome to whistleblowing. Illustrating this, Luke said: “I know that they [the organisation] are on my side and that I would not be silenced by them … and if this is the case, then I would directly address a commission and communicate it there” whilst Lee indicated that “the leadership are all against it. It would definitely be the decision of the athlete, and no one would defend him at all, or come to his defence”. In line with the predictions of TPB (1), we see evidence that an anti-doping culture increases the likelihood of whistleblowing as athletes are confident that the behaviour will have a desirable consequence. The provision of organisational support and protection serves to mitigate or reduce perceived costs by enhancing an individual's expectation that whistleblowing will have a positive outcome. Furthermore, the extent to which the sporting organisation values and promotes anti-doping behaviour appears to represent a crucial determinant of external whistleblowing.

Similarly, Scott suggested that he would “personally be more comfortable having a conversation if they [the doper] were aware of anti-doping rules and regs and they [the organisation] would happily rather not see someone use drugs”. Athletes who were morally opposed to doping wanted to see their organisation take a similarly principled stance. Sharing the same set of principles is an important factor in the decision to whistleblow as revealed by Mike:

*They have the same principles around the situation. Therefore, you'd feel more comfortable disclosing it to them, whereas if you had an inkling that the coaches and other athletes in the team are more relaxed about the situation, would let it slide as such, then I would definitely feel less comfortable in bringing up that situation*.

Sharing these anti-doping values also increases the trust the athlete has that their organisation will deal with a complaint in an appropriate manner as illustrated by Mike: “if I believe that the club's got the right culture and ethics then I’ll probably have more trust in them dealing with the situation … a culture where it's prohibited and everyone has the same ethos and values”. This statement provides further evidence of the important role played by what Ajzen (1) referred to as “non-motivational factors” (including the availability of requisite opportunities and resources) in determining whether athletes feel that a successful outcome to whistleblowing is under their volitional control. In this case, the cooperation of others, which includes sharing a similar set of values, may increase athletes' *actual* control over whistleblowing (1). The findings also suggest that the subjective norms held by the leaders in their organisation would play an important role in their decision to come forward, as articulated by Mike:

*the type of culture and the ideology that the leader holds … if I felt that I was going to approach the leadership team and say “oh, I believe athlete x is doing this” but their ideology and culture around doping is that it should be allowed then I'm not going to approach him because I'm just going to get shot down straight away and then it makes me look like I'm trying to target the individual*.

It was also important that leaders clearly and openly articulate their stance on doping as suggested by Luke: “there simply has to be a clear anti-doping structure in the leadership that is communicated in an open manner”. Operating within a clear anti-doping structure may increase the athlete's perception of the ease of reporting wrongdoing and strengthens their conviction that they will be protected [see (26)]. Again, the intention to whistleblow will not manifest in actual behaviour unless athletes have the required opportunities and resources (1) to successfully do so.

In contrast, a lack of support from leaders, or a sense that they were ambivalent about doping, would reduce the likelihood of participants exposing this practice as noted by Paul:

*I'd potentially turn a blind eye unless I knew I had 100% support from hierarchy. And it was not going to get directly related back to me … if all the team administration, some of the players and majority of people were supportive of flagging it, then I would be happy to do so*.

It was also important for the athletes that they could trust that leaders would take appropriate action (i.e., confront the doper and protect the whistleblower) as some were afraid that the allegation might be downplayed, ignored, or swept under the carpet. The existence of a strong anti-doping culture, exemplified by leaders who were morally opposed to doping, was essential if participants were to feel confident that they would be protected in the event that they blew the whistle externally.

### Social support

Those participants who emphasised the importance of team climate within their organisation also considered the social support they received from colleagues and coaches as playing a central role in the whistleblowing process. Marcus argued that:

*It purely depends on the support of your coach, club, teammates and club administration in terms of how do I feel as a member of a particular organisation and especially in team sports. The idea of unity, of one cohesive group of players that is all on the same page about everything. How the organisation is being run is the most crucial component I personally think in terms of the reaction of particular players within the organization. If you have a great relationship with them you go to them…because you are concerned about the team*.

The support they receive from colleagues thus represents another important “non-motivational factor” that increases confidence that successful whistleblowing is within the athlete's volitional control (1). Indeed, a number of participants lacked the confidence to act on their suspicions of doping unless they had the full support of their teammates and/or coaches. To illustrate, Mike acknowledged that:

*It's hard without damaging relationships. But if I get an indication that another teammate suspected the same thing, if I can subtly get the indication that they're thinking the same thing. And develop the trust that I can say to my teammate “are you thinking the same thing?”… And shift that personal responsibility onto a collective responsibility. Then I’d be more likely to report it … it's having the support of other individuals. If I was confident that 7 other individuals would stand by me, would stand by each other as a collective when reporting this incident, I would feel more comfortable because that removes the possibility that you're going to lose them social interactions. You're going to lose that trust*.

Ava also noted that a lack of social support would lead to an unfavourable attitude (1) towards whistleblowing:

*it would certainly be easier to report an incident if I had the support of the people who run the club. If the people who run the club told me not to report doping, I would be in big trouble knowing what the consequences would be for me*.

Athletes placed particular emphasis on the support they receive from their coach as a crucial determinant of the intention to whistleblow as revealed by Eliza:

*It would definitely be easier for me if I have the support of my coach. The support of my coach means a lot to me. My thinking and my views on doping actually come from him because he taught me all about it. He taught me how wrong everything is about doping and how bad it is for our health and then for sports in general*.

Luke hinted at the importance of subjective norms within his sport and the likelihood that his fellow athletes would support his decision to blow the whistle: “it should be solved with other tools in a reasonable manner so that as many athletes and other people involved in the process, not just the athlete who consumed the substances”. This sense of collective action or support may prove re-assuring as it is likely to provide the athlete with a degree of protection from potential recrimination. In expressing this point, Paul argued that he'd:

*be more likely to report it providing I had the support and backing from other people to do so. If you've got strong athletes that are willing to stand up and say what they believe in then I'd be more than happy to do the same. I'd happily raise anti-doping allegations if I knew I had strong support from the team, athletes and staff*.

Athletes who felt they would receive this level of support from their teammates would be more inclined to approach the authorities with a complaint. However, in deciding whether or not to pursue a whistleblowing complaint, a number of athletes also appeared to engage in a cost-benefit analysis including a consideration of the type and length of ban the PED user might receive.

### Facilitating factors

Analysis highlighted a number of facilitating factors for whistleblowing. These included the assurances that athletes would receive from the authorities and the extent to which their anonymity would be preserved should they decide to report doping. These findings build on Erickson et al.'s (25) work by showing how athletes’ responses to witnessing PED use will differ dependent on context.

### Assurances

Participants needed assurances that their accusations would be treated with considerable discretion and that the authorities would launch a thorough investigation. Paul explained:

*you want to know that if you're going to report it it's going to be treated seriously and with the strictest confidence and it's going to be looked into. You just don't want it to be swept under the carpet and to no avail*.

Participants were willing to whistleblow on the condition that accusations would be dealt with seriously and processed via appropriate channels as evidenced by Oscar who suggested: “I mean I want to be certain that the topic is addressed to the right people and not washed through a third person or that some damages might occur”. Processing a complaint via these channels was the best way of ensuring that it would be treated with care and discretion as discussed by Luke:

*But of course they have to be verified parties, to which I could report, so that I can be sure that those parties are safe. I have to be sure that my concerns will be treated in the right way and that something happens with it … if it would just result in a warning I would not see the sense in reporting it and risking something. But if I would know that the athlete would be kicked out of the sport club and banned from competitions for a certain time, this would have an impact*.

To mitigate against perceived costs that might arise from whistleblowing, athletes wanted assurances that an appropriate level of punishment would be meted out to the PED user should the authorities be informed of a doping offence and the athlete subject to an ADRV. Participants were adamant that dopers should receive a lengthy ban and many of them expressed reluctance to whistleblow if they suspected that the authorities would treat the doper leniently. Luke felt that if reporting an offence merely resulted “in a warning, I would not see the sense in reporting and risking something and be put in a bad position”. For Andrea, reporting a doping offence must mean “that someone who doped cannot take part anymore. That he or she is excluded from the sport. Or at least he/she will be ineligible to compete” whilst Oscar added:

*I hope that if one would report it, that consequences will definitely follow … the main outcome you would hope for is obviously a four or two year ban depending on the severity … a four-year ban is justified so why not disclose it? I think that individual deserves that punishment*.

If the PED user receives a lengthy ban then the whistleblower avoids the rather daunting prospect of encountering that athlete for some time. Whistleblowers may also feel that such a strong ban reinforces the severity of the offence and provides further justification for their decision to report it in the first place. Together, these factors increase the likelihood that they would come forward with a whistleblowing complaint.

### Anonymity (preference for 3rd party reporting)

A number of athletes who suggested that they might be prepared to blow the whistle externally admitted that they were unaware of where or how they would report a suspected doping offence. This uncertainty is reflected in other studies exploring whistleblowing in sport [see 26)]. Generally speaking, participants who were prepared to disclose information would prefer to do so to someone outside their immediate environment thereby reducing the likelihood that their identity might be uncovered. Some wished to avoid raising suspicion by being seen, as Paul puts it, “going directly to the coach to have a sit-down face-to-face meeting where people could see that”. Instead, some athletes would consider whistleblowing if they could report their concern to a third party. Paul noted that he “wouldn't flag it with the club, I’d be going higher up to a different channel” whilst Andrea suggested that “if there would be another third party, from which I know that it is a relevant institution … it is definitely helpful … This person is independent of my sport and hopefully objective, and this is important to me”.

Reporting the incident to a third party provides athletes with a level of protection that they may not be afforded if they reported doping “in-house” (i.e., within their own organisation or within the sporting federation). In fact, some expressed doubts that their own sporting federation would handle the matter appropriately. Illustrating this, Lee revealed that:

*no federation wants a doping scandal. I do not know if, in some federations, this results in the opposite direction … meaning that you are muzzled, instead of it being forwarded. I would rather let an independent organisation handle this situation”*.

The stigma of approaching someone in-house was also revealed in Erickson et al.'s (26) study where athletes reported a lack of trust in their sporting federations ability to properly manage or “take care” of their complaint. There was some support, however, for reporting via anonymous phone lines. Mike, for example, acknowledged that “the higher up I can go … straight to WADA … the more traceability it removes” whilst Luke thought that:

*a telephone call can lead to fast solutions, because you can directly ask further questions, as opposed to writing a letter, which has to be sent back to me for further questions and it's not anonymous anymore. With a telephone call they can ask follow-up questions so that a lot of information can be provided*.

It is important to note, however, the difficulty of establishing a consensus amongst the group as to their preferred mode of reporting. Whilst many expressed a preference for third party reporting, others remained concerned about whether their anonymity could, in fact, be preserved. Mike, for example, saw the advantages afforded by a telephone call but also thought that “an anonymous phone line still has an element of traceability”. Luke mentioned his concerns logging an incident with a website given that “the IP address could reveal information for tracing”. These findings suggest that authorities will struggle to increase the likelihood of whistleblowing until they can guarantee athletes that various modes of reporting will preserve their anonymity.

Whilst anonymity was crucial for many participants, several of the athletes were so strongly opposed to doping that they had little concern whether or not their identity as a whistleblower was preserved. Lee disclosed that “it [anonymity] would not really influence me, whether the teammates are friends or not, because in the moments they do such things, they are not friends anymore”. Overall, the findings elucidate the complex nature of the reporting process and the challenges faced by authorities in convincing athletes that their anonymity will be preserved and that they will be protected from potential recrimination.

## Discussion

The current study contributes to a growing body of literature [e.g., (26),] that shows that whistleblowing is a dynamic process characterised by the interaction of a range of personal, organisational, and psychological factors in determining the intention to report PED use. Our research substantiates Erickson et al.'s (6, 25, 26) finding that confrontation represents athletes' preferred option for dealing with doping. However, our findings extend Erickson et al.'s (6, 26) work by identifying organisational factors that influence the likelihood that athletes would move beyond this initial confrontation by whistleblowing through official channels. Many of the athletes who were prepared to whistleblow externally were confident that they would be protected from retaliatory action. These athletes appear to operate in sporting organisations characterised by reciprocity and social support and this has been found to increase the likelihood of whistleblowing behaviour in other studies [see (23, 38)]. The level of support they receive from coaches or other authority figures had a significant bearing on the confidence athletes placed in the whistleblowing process. This mirrors findings from the organisational literature which has shown that an individual's trust in their supervisor mediates the relationship between organisational justice and the intention to whistleblow (39). Athletes who were prepared to whistleblow possessed a greater understanding of, and commitment to, the espoused values of their in-group and this increases their inclination to protect their sporting sub-culture by exposing wrongdoing [see (38)].

Although moral reasoning is typically thought to be an important motivator for confronting doping [see (9, 25, 26)], this sense of right and wrong would not automatically compel some of our athletes to blow the whistle externally. Instead, in line with the TPB, these participants weighed up the availability of opportunities and resources that would allow them to successfully perform the desired behaviour. Consistent with Erickson et al.'s (6, 25) findings, many athletes' preferred course of action upon learning of doping behaviour is to confront the suspected doper directly or to disclose their concerns to coaches in the hope that the matter could be addressed without the need to involve the authorities. Erickson et al. (6) argued that athletes confront PED users as a form of threat, thereby providing them with a chance to change their behaviour. Confrontation is thought to offer a viable strategy for discouraging doping as it represents a communal effort that takes advantage of everyday behaviours as a deterrent (6). Confrontations are seen as an everyday form of regulation that might deter doping through “informal sanctions” from significant others (6).

Whilst confrontation offers promise as a deterrent to doping, for many of the participants who were reluctant to blow the whistle externally, the desire to observe a code of silence and remain loyal to their teammates seems to outweigh any sense of moral responsibility to report doping to the relevant authorities (35). Those athletes who revealed they would not go to the authorities do so on the basis of a cost-benefit analysis which results in them concluding that the perceived costs of whistleblowing externally are far greater (e.g., being ostracised by their teammates) than those that might arise from keeping the matter “in-house”. The findings point to a series of “intervening events” (1) including a perceived lack of organisational protection (e.g., ethical leadership) within some sporting sub-cultures, which present an important obstacle to whistleblowing. Cost-benefit calculations have been found to be a more important motivator for internal rather than external whistleblowing as the analysis can be more accurately applied in a situation which is stable and in which one can predict results with a greater degree of certainty (40). The finding that few athletes were aware of the reporting mechanisms available, and had concerns that their complaints would be handled improperly, merely compounds the latter quandary.

Future research may wish to explore how cultural factors influence the importance athletes attribute to loyalty or fairness (see (3) and (41), for some initial findings on this topic). Given that loyalty has been suggested to be a more dominant norm within collectivist cultures than individualistic ones, large-scale quantitative studies could explore whether this is associated with the increased likelihood that athletes will overlook unethical acts (17, 18). Our findings cast some doubt on the oft-cited argument that offering rewards and positive recognition to whistleblowers will increase this practice. The shame or guilt some athletes are likely to feel for betraying their teammates (i.e., if they adhere to loyalty norms) will outweigh any perceived reward these athletes might gain from whistleblowing. Perhaps a more parsimonious approach to increasing the likelihood of whistleblowing is to persuade athletes that doping represents a significant threat to the in-group's best interests. In Waytz et al.'s (18) study, for example, participants primed to endorse fairness reported more willingness to blow the whistle than those primed to endorse loyalty. Of course, this may only prove successful if there are wider attempts by sporting organisations to address the problem of doping. Nevertheless, educators may wish to emphasise that whistleblowing will contribute to the *greater good* of the athlete's sport thereby positioning this behaviour as representative of a “larger loyalty” (42). Another potentially fruitful approach is to encourage athletes to confront athletes within their in-group by expressing their dissent when they witness or become aware of unethical behaviours (43). One can engage in constructive dissent, whilst continuing to privilege group loyalty, by emphasising how specific behaviours are harmful to the team or not in the best interest of one's sport. According to Dungan et al. (43), this approach might prove effective because “people who have dissenting opinions that benefit the group may be rewarded and viewed as effective leaders” (p. 131).

A potential limitation of our study is that we explored the intention to whistleblow rather than actual instances of real-world whistleblowing behaviour. However, a systematic review of whistleblowing behaviours in a wide range of disciplines including nursing and the armed forces, found considerable similarities between how whistleblowers would deal with hypothetical and actual scenarios (9).

Our findings that organisational support and protection decrease perceived personal costs, and increase whistleblowing intentions, have several implications for the education and promotion of whistleblowing. Whistleblowing policies should be embedded within a sport's organisational structure and management teams should explicitly communicate the organisation's mission in the field of whistleblowing. The latter strategy is an important step because athletes may be more forthcoming with information if whistleblowing processes are open, transparent, and positioned as central to a group's collective goals. In addition, NADO's might improve athlete trust in the whistleblowing process by publishing examples of cases in which they have resolved claims of wrongdoing. Training could be provided for coaches on the best ways to respond to reports of doping and how they might support athletes through the entirety of the whistleblowing process. Finally, education is also required to make athletes aware of the availability of legal protection for whistleblowers and the various reporting mechanisms at their disposal (17).

To conclude, we should be careful not to place too great a reliance on whistleblowing as a means of identifying doping in sport given the difficulty NADO's face in convincing athletes that whistleblowing will have a positive outcome. That said, our findings hold promise as they suggest that the promotion of fairness norms that emphasise a group's collective interests might encourage athletes to view whistleblowing as a means of increasing group cohesiveness and effectiveness and thereby increase the likelihood of this practice.

## Data Availability

The original contributions presented in the study are included in the article/Supplementary Material, further inquiries can be directed to the corresponding author.
